# Endothelial CD34 expression and regulation of immune cell response in-vitro

**DOI:** 10.1038/s41598-023-40622-7

**Published:** 2023-08-19

**Authors:** Lousineh Arakelian, Julien Lion, Guillaume Churlaud, Rezlene Bargui, Briac Thierry, Evelyne Mutabazi, Patrick Bruneval, Antonio José Alberdi, Christelle Doliger, Maëva Veyssiere, Jérôme Larghero, Nuala Mooney

**Affiliations:** 1 Human Immunology, Pathophysiology, Immunotherapy, Inserm UMR 976, Paris, France; 2 Université Paris Cité, Paris, France; 3 CIC de Biothérapies CBT 501, Paris, France; 4 Unité de Thérapie Cellulaire, AP-HP, Hôpital Saint-Louis, Paris, France; 5 AP-HP, Hôpital Saint-Louis, Centre MEARY de Thérapie Cellulaire et Génique, 75010 Paris, France; 6 Service d’ORL Pédiatrique, AP-HP, Hôpital Universitaire Necker, 75015 Paris, France; 7 Service de Cardiologie, Hôpital Européen Georges Pompidou, 75015 Paris, France; 8 UMS Saint-Louis US53/UAR2030, Université Paris Cité - INSERM – CNRS, Institut de Recherche Saint Louis, Paris, France

**Keywords:** Cytokines, Cell biology, Stem cells

## Abstract

Endothelial cells cover the lining of different blood vessels and lymph nodes, and have major functions including the transport of blood, vessel homeostasis, inflammatory responses, control of transendothelial migration of circulating cells into the tissues, and formation of new blood vessels. Therefore, understanding these cells is of major interest. The morphological features, phenotype and function of endothelial cells varies according to the vascular bed examined. The sialomucin, CD34, is widely used as an endothelial marker. However, CD34 is differentially expressed on endothelial cells in different organs and in pathological conditions. Little is known about regulation of endothelial CD34 expression or function. Expression of CD34 is also strongly regulated in-vitro in endothelial cell models, including human umbilical vein endothelial cells (HUVEC) and endothelial colony forming cells (ECFC). We have therefore analysed the expression and function of CD34 by comparing CD34^high^ and CD34^low^ endothelial cell subpopulations. Transcriptomic analysis showed that CD34 gene and protein expressions are highly correlated, that CD34^high^ cells proliferate less but express higher levels of IL-33 and Angiopoietin 2, compared with CD34^low^ cells. Higher secretion levels of IL-33 and Angiopoietin 2 by CD34^high^ HUVECs was confirmed by ELISA. Finally, when endothelial cells were allowed to interact with peripheral blood mononuclear cells, CD34^high^ endothelial cells activated stronger proliferation of regulatory T lymphocytes (Tregs) compared to CD34^low^ cells whereas expansion of other CD4^+^-T cell subsets was equivalent. These results suggest that CD34 expression by endothelial cells in-vitro associates with their ability to proliferate and with an immunogenic ability that favours the tolerogenic response.

## Introduction

Endothelial cells (EC)s are cells that form the lining of blood vessels. Most of these cells originate from the mesoderm during embryogenesis and share common progenitors with cardiac and hematopoietic cells^1^. The main functions of ECs are: to ensure the fluid circulation of blood and lymph through the blood vessels and lymphatic nodes, the regulation of hemostasis after injury, activation or inhibition of immune cells as well as the regulation of transendothelial migration of circulating cells into the tissues and sites of injury^2^.

ECs have different characteristics according to their localization. Such differences are believed to promote their different functions according to the size of the vessel and the organ in which it is located. As an example, in arteries where blood pressure is high, ECs are elongated and form much tighter intercellular junctions compared with venous ECs that are exposed to lower blood pressure. Post-capillary venules are the primary site of leukocyte trafficking and ECs form less well organized and therefore less tight junctions in such venules^3^. Other ECs, such as sinusoidal cells in the liver and bone marrow, or glomerular ECs present fenestrations that are related to their functions in hematopoiesis and/or filtering blood molecules^4–6^.

Regarding expression of cell surface markers, all ECs share a number of common markers including Platelet endothelial cell adhesion molecule 1 (PECAM-1) also known as CD31, endoglin or CD105, Vascular endothelial (VE)-cadherin (CD144). Expression of other EC markers is regulated in different organs or by vascular injury. For example, in the context of organ transplantation, where the microvascular endothelium is the first site of interaction between the allograft and the graft recipient, ECs are a target for alloantibody mediated damage leading to allograft dysfunction. Increased endothelial expression of major histocompatibility complex class II molecules (MHC II) and cell adhesion molecules^7^ is observed in response to inflammation and indeed inflammation constitutes an independent risk factor in antibody-mediated rejection.

The single-pass transmembrane sialomucin protein CD34 is a surface marker with regulated EC expression ranging from high to low. CD34 is expressed on early hematopoietic and vascular-associated tissue^8^. It is encoded by a gene located on the long arm of chromosome one^9^ and is widely documented as a stem cell marker for different cells such as hematopoietic, muscle satellite and epithelial progenitors^10^.

In human endothelial vascular beds in-vivo, CD34 is strongly expressed in biopsies of larger blood vessels such as arteries and veins, as well by smaller capillaries^11^. On the other hand, very little or no expression has been detected in the liver or spleen sinusoids and marginal sinuses of lymph nodes. In some pathologies, including liver fibrosis or cancers, endothelial expression of CD34 is modified and it has been proposed as a marker of progression of such diseases^12,13^. It has been reported that CD34 is a ligand of L-selectin, expressed by circulating blood cells and involved in cell trafficking through lymph nodes^14^. However, the role of CD34, its regulation and the importance of newly expressing CD34 cells in different pathologies is not fully understood.

As for *ex-vivo* studies, when human umbilical vein endothelial cells (HUVEC) were isolated and amplified under 2D culture conditions, CD34 expression was lost by a fraction of ECs. It was previously demonstrated in HUVECs that CD34 strongly correlates with high cell density and low proliferation, and that CD34 expression decreased with successive cell passages^15^. In a comparative study of CD34^+^ versus CD34^−^ HUVEC, Siemerink and al. reported that CD34 expression was reversible, that CD34^+^ were less proliferative and had a higher expression of genes typical of tip cells^16^. Others have reported that CD34^+^ ECs correspond to tip cells and have greater ability to form tubes in fibrin^17^. Furthermore, CD34 expression was regulated and reduced by inflammatory cytokines such as tumor necrosis factor-a (TNF-α), interferon gamma (IFNγ) and Interleukin 1 (IL-1) in-vitro*,* and this regulation was inversely correlated with that of adhesion molecules such as Intercellular adhesion molecule-1 (ICAM-1) and E-selectin^15^.

Despite such studies on CD34 expression in ECs, little is known about cells expressing high versus low levels of CD34^+^, and particularly regarding their immunogenicity. We have therefore investigated CD34^high^ HUVECs and CD34^high^ endothelial colony forming cells (ECFCs) in-vitro.

## Materials and methods

### Endothelial cell models

For this study, two models of ECFCs and HUVECs were used.

### ECFC isolation and culture

ECFCs were isolated in our laboratory from umbilical cord blood. Healthy donors’ cord blood was obtained from Saint-Louis Hospital Cord Blood Bank (registered to the French Ministry of Research under number AC-2016-2756 and to the French Normalization Agency under number 201/51848.1). Peripheral Blood Mononuclear Cells (PBMC) were isolated by centrifugation on Lymphocyte Separation Medium (Eurobio Scientific), and were plated in 6 well culture dishes (Falcon, Corning, USA) coated with collagen I (Collagen Type I Solution from rat tail, Sigma-Aldrich, USA) at a density of 1 × 10^5^ cells/cm^2^, in EGM2-mv culture medium (Lonza, USA). The medium was renewed twice a week, until the appearance of adherent cell colonies.

Cells were then detached by trypsin (0.25% Trypsin-EDTA 1X, Gibco, UK) treatment and reseeded in 75 cm^2^ culture dishes for amplification. Cryopreserved cell banks were prepared. For the following experiments, four different donors were used.

### HUVECs

Cryopreserved HUVECs were obtained from Lonza. They were cultured at a density of 5 × 10^3^ cells/cm^2^, in EGM2-mv culture medium. The medium was renewed twice a week. Cells were amplified and detached by trypsin treatment. A cryopreserved working cell bank was prepared after thawing. For the following experiments, two different batches were used.

### Cell characterization by flow cytometry

The endothelial profile of the isolated and cultured cells was evaluated by flow cytometry, after staining with the following antibodies (Table 1):

**Table 1:** Antibodies used for endothelial cell characterization by flow cytometry.

Antibody	Reference
Mouse IgG1-FITC (BD)	555909
Mouse IgG1-PE (BD)	345816
Mouse IgG1-APC (BD)	345818
FITC Ms anti human CD31 (BD)	555445
Anti-h CD105/Endoglin PE (R&D)	FAB10971P
CD144 Alexa 647 (BD)	561567
CD34-PE (Miltenyi Biotec)	130-081-002

Staining was performed with 2µl of antibody/ 1 × 10^5^ cells for 30 min at 4 °C. Analysis were carried out on an Attune-NxT (Thermo Fisher Scientific) flow cytometer.

### Magnetic cell sorting of CD34 ECs

HUVECs and ECFCs were amplified in Cell-STACK plates (Corning). Highly confluent cells were detached with trypsin and stained with CD34 MicroBead kit (Miltenyi Biotec, Germany) for 30 min at 4 °C. Cells were rinsed and suspended in PBS/EDTA 2 mM. Cell sorting was then performed on LS columns (MACS Miltenyi Biotec) using a Manual MACS® Magnetic Separator (MACS Miltenyi Biotec). Sorting efficiency was assessed by flow cytometry, after staining with CD34-PE (Table 1). Analyses were done on Attune-Nxt cytometer.

### Kinetics of CD34 expression in sorted cells

To evaluate the effect of cell confluency on CD34 expression, two series of experiments were performed. Firstly, CD34^high^ cells were reseeded at a density of 2 × 10^5^ cells/cm^2^. CD34 expression was monitored over time, up to 18 days.

In a second experiment, CD34^high^ cells were seeded at densities of 5 × 10^4^ and 1 × 10^4^ cells/cm^2^, in a 6 well culture plate (n = 3 wells/condition). CD34 expression was then evaluated at day 5.

Cells were stained by anti CD34-PE as and were analyzed by flow cytometry, as described above.

### Immunohistology of the umbilical vein

To evaluate the endothelial marker expression and proliferation, a segment of an umbilical cord was obtained from Saint-Louis Hospital Cord Blood Bank. The umbilical cord was collected from a healthy donor, according to the European Directive 2004/23/EC, after written informed consent had been obtained and serological testing carried-out according to national regulatory agency requirements. A full-term delivery cord was collected and processed within 24 h. It was fixed in paraformaldehyde 4%, embedded in paraffin and 5µm thick sections were prepared. The samples sections were then stained labeled with anti-CD31 (Dako-Agilent, Les Ulis, France), anti-CD34 (Dako-Agilentref) and anti-Ki67 antibodies (Dako-Agilentref).

### Fluorescence activated cell sorting of CD34 cells for transcriptomic studies

For these experiments, ECFCs were obtained from four donors. Cells were amplified at a large scale in Cell-STACK plates (Corning). At high confluence, cells were detached using trypsin, rinsed in PBS and EDTA 2mM (Euromedex, France), passed on cell strains of 40 µm pores (Falcon, Corning, USA) and then stained with anti-CD34 antibody. CD34^high^ and CD34^low^ fractions were then sorted on an Aria II cell sorter. Immediately after sorting, cells were centrifuged, rinsed in PBS and frozen in Buffer RLT Plus (Qiagen, Germany).

### Comparative gene expression study

For this study, CD34^high^ and CD34^low^ sorted ECFCs were obtained from four different donors. However, due to heterogeneity of CD34 expression, two donors were used for the preparation of both CD34^high^ and CD34^low^ fractions, one donor for only CD34^high^ (due to a very high expression of CD34) and another for only CD34^low^ (due to a very low expression of CD34) fractions. In all, four different donors were used to prepare three CD34^high^ and three CD34^low^ samples. RNA was extracted using the RNeasy Plus Mini Kit (Qiagen, Germany), according to the manufacturer’s instructions. RNA quantification and quality control was performed using the HT RNA Reagent Kit and the Caliper LabChip Microfluidics System (Perkin Elmer). One hundred nanograms of total RNA was amplified, labeled, and fragmented using GeneChip Plus Reagent Kit (Thermo Fisher Scientific). Each sample was hybridized onto GeneChip® Human Transcriptome Array 2.0 (Thermo Fisher Scientific), washed, and stained with the Affymetrix® Fluidics Station 450. Array Scanning was performed with the Affymetrix® GeneChip Scanner 3000 7G using the Command Console software (Thermo Fisher Scientific) and then analyzed using the Affymetrix® rma-sketch routine. Differentially expressed genes (DEGs) were identified using Limma R software package^18^. The False Discovery Rate (FDR) adjusted *p* value of each coding gene was calculated by applying the Benjamini–Hochberg procedure to the differential analysis *p* value. The fold change (FC) of every gene, together with their corresponding FDR adjusted *p* value, was used for selection of DEGs. These values were calculated for coding genes and we did not consider the non-coding probes in our analysis.

### Validation of transcriptomic results by RT-qPCR

#### Reverse transcription (RT)

RNAs of the magnetic sorted cells were extracted using the RNeasy Plus Mini Kit (Qiagen, Germany), For each sample, 4 µg pf RNA was reverse-transcribed to cDNA using the High capacity cDNA reverse transcription kit (Applied Biosystems, Lithuania) according to manufacturer’s instructions. RT was carried out in 0.5 mL tubes (Easy strip Snap Tubes, Thermo Fisher Scientific, UK) in a SimpliAmp Thermocycler (Applied Biosystems), using the following program: 10 min at 25 °C, 120 min at 37 °C, 5 min at 85 °C, followed by a cooling step at 4 °C.

#### Quantitative PCR (qPCR)

The cDNAs were diluted to 1/50 in nuclease free water and were used for qPCR. TaqMan™ probes were used for the amplification. The reactions were carried out in 96 well reaction plates (Applied Biosystem, China) in a final volume of 25 µL, with 5 ng cDNA per well. Genes previously identified by the transcriptomic study were evaluated. The housekeeping gene that was retained after a screening phase of different genes (not shown) was RPLP0.

The following Taqman probes were used for the qPCR experiments (Table 2).

**Table 2:** TaqMan probes used for qPCR of endothelial cells.

Gene name	Assay ID
RPLP0	HS99999902_m1
CD34	Hs02576480_m1
CENPE	Hs01068241_m1
ANGPT2 (Angiopoietin 2)	Hs0148042_m1
IL-33	Hs01125943_m1
IL1RL1 (ST2)	Hs00249384_m1

Results were analyzed using the Quant Studio 7 software. Relative quantity (RQ), as well as RQ Max and RQ min (representing the upper and lower limits respectively) were exported. Graphs were prepared using Graph Pad PRISM. In our study, a difference of RQ by a minimum factor 2 was considered as significant, with a 95% confidence interval.

### Co-culture of ECs with PBMCs

**Figure 1:**
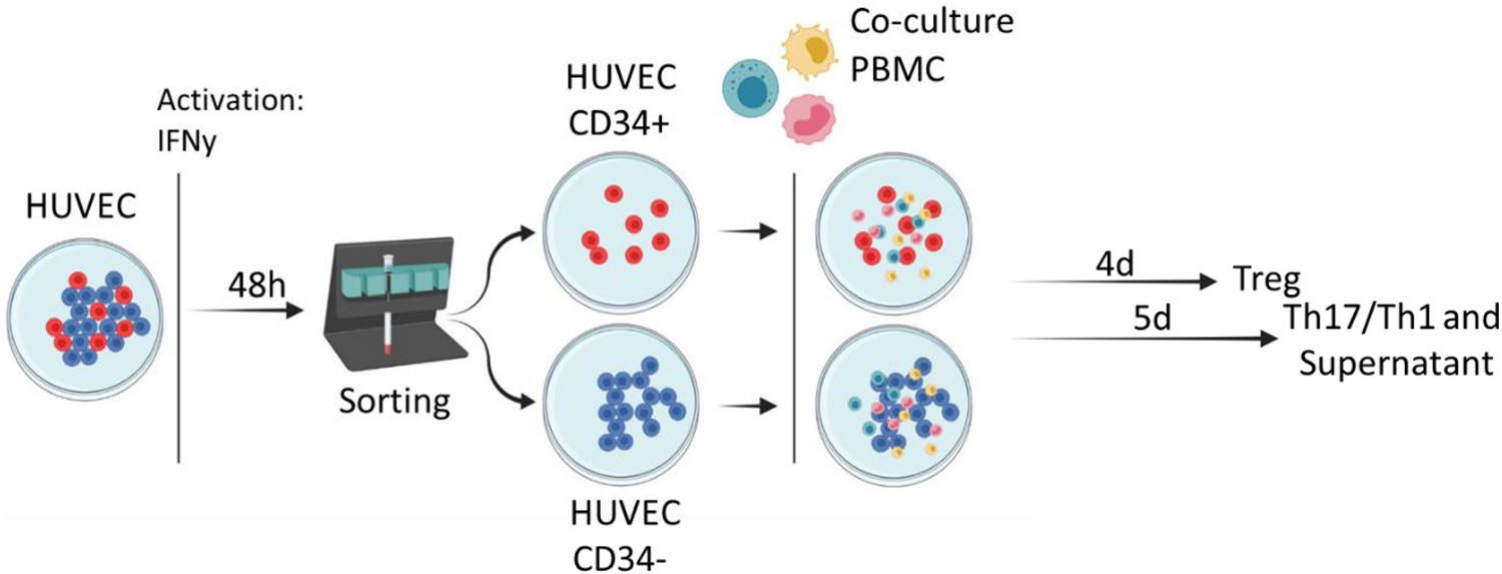
Protocol of cell sorting of HUVEC for co-culture with PBMCs.

We then evaluated the immunogenicity of CD34^high^ and CD34^low^ ECs in allogenic cultures with non-HLA-matched PBMCs. These experiments were carried on two different batches of HUVEC. Cells were cultured in Cell-Stack plates to high confluence and then IFN-γ (Biolegend) was added at a concentration of 30 ng/ml for 48 h in order to increase HLA class II expression and their interaction with lymphocytes (as described previously^19^). Cells were then seeded at a density of 5 × 10^4^/well in a 96 well culture plate (Falcon) to adhere overnight at 37 °C in complete RPMI medium (GIBCO, Thermo Fisher Scientific) supplemented with 10% fetal bovine serum (FBS) (Eurobio) and a final concentration of HEPES 10 mM, sodium pyruvate 1X (Eurobio), glutamine 2 mM, penicillin 100 IU/mL and streptomycin 100 μg/mL (GIBCO, Thermo Fisher Scientific). The next day, the culture medium was renewed and PBMCs were added at a ratio 1:1 in complete RPMI medium (Fig. 1).

Supernatants from co-cultures were collected after 5 days for soluble factor quantification. Proliferation of regulatory T lymphocytes ((Treg), the CD4^+^CD45RA^−^FoxP3^bright^CD127^low^ subpopulation), was assessed in Carboxyfluorescein succinimidyl ester (CFSE)–labeled PBMCs after 4 days of co-culture.

The Th17 and Th1 CD4^+^-T subpopulations were assessed after 5 days of co-culture. PBMCs were stimulated by phorbol-12-myristate-13-acetate (PMA) 50 ng/mL and ionomycin 1 μM (Cell Signaling Technology) with GolgiStop 1 × (BD Biosciences) for 4 h, and Th17 (CD3^+^CD8^−^IL-17^+^) and Th1 (CD3^+^CD8^−^IFNγ^+^) subpopulations were analyzed by flow cytometry using the following antibodies (Table 3):

**Table 3:** Antibodies used for the characterization of lymphocyte populations.

Antibody	Reference
CD4-APC (BD Pharmingen)	555349 (RPA-T4)
CD25-PE (Biolegend)	356134 (M-A251)
CD127-BV510 (Biolegend)	351332 (A019D5)
FoxP3-BV421 (Biolegend)	320124 (206D)
CD45-PECy7 (Biolegend)	304126 (HI100)
CD3 PerCP (Biolegend)	344814 (SK7)
CD8 Pacific blue (Biolegend)	344718 (SK1)
CD4 PE (Biolegend)	300508 (RPA-T4)
IL-17 A647 (Biolegend)	512310 (BL168)

### ELISA

IL-33, soluble ST2 and Angiopoietin 2 secretion was evaluated in culture supernatants of sorted HUVECs alone or in co-culture with PBMCs. Supernatants were collected on day 5 and IL-33, ST2/IL-33R and Angiopoietin 2 (Quantikine ELISA Kit (all from R&D systems)), were determined according to the manufacturer instructions.

## Results

### Morphological and Phenotypic characterization of ECFCs and HUVECS

**Figure 2:**
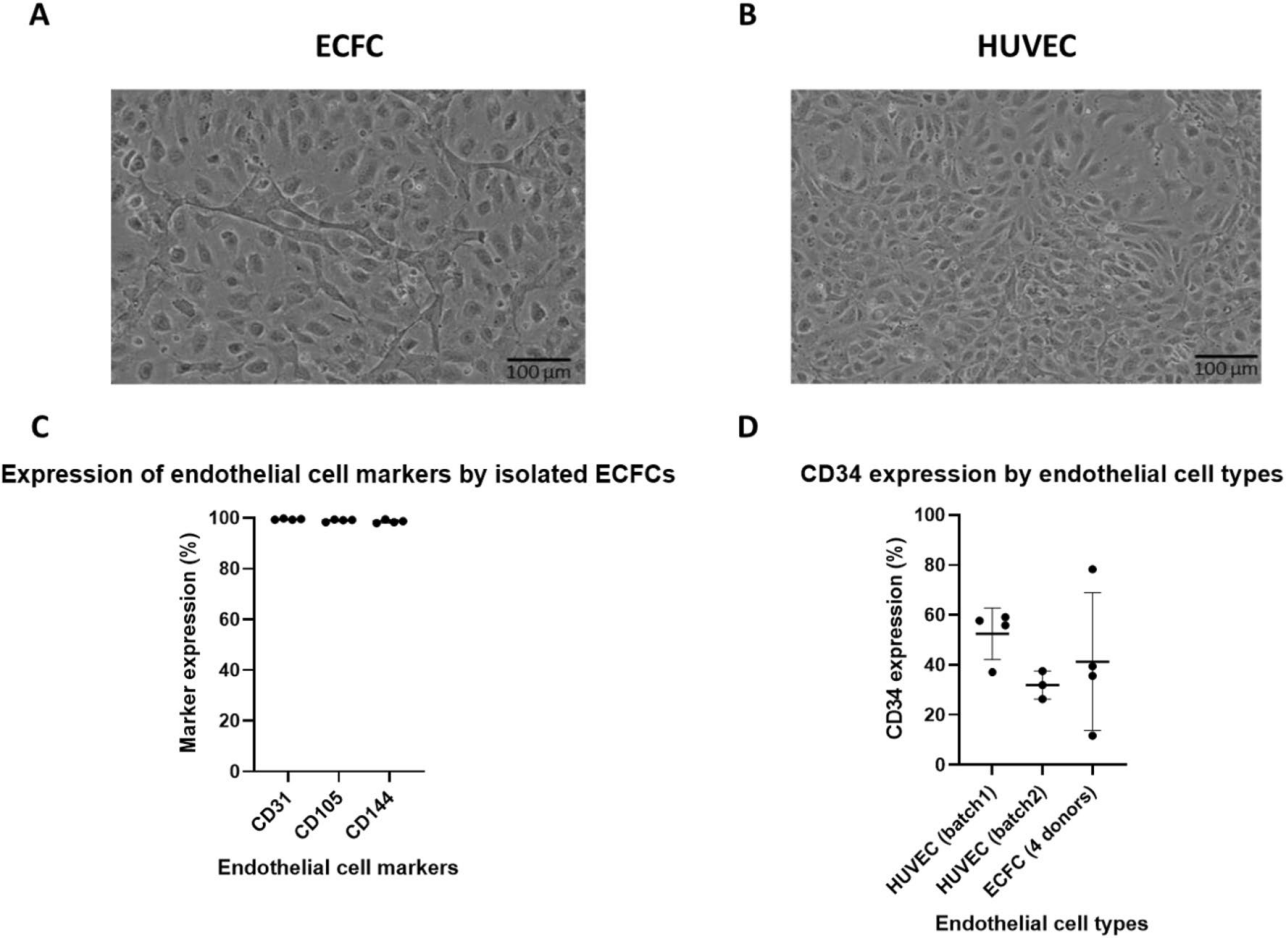
Morphological and phenotypic characterization of ECFCs and HUVECs. ECFCs (**A**) and HUVECs (**B**). Evaluation of characteristic endothelial markers CD31, CD105 and CD144 from four different ECFC donors (**C**). CD34 expression in two different batches of HUVEC and four donors of ECPCs (**D**). No significant statistical difference was observed between these cell types (One-way ANOVA, p value 0.3780). However, a large variability was observed between the donors of EFCFs. All flow cytometry experiments were performed at high confluence.

ECFCs and HUVECs had similar morphology, composed of elongated cells in culture (Fig. 2A,B). In confluent populations, some size heterogeneity was observed in both cell types.

ECFCs strongly expressed endothelial markers CD31, CD105 and CD144 (Fig. 2C). Similar results were obtained with HUVECs. The CD34 expression varied in ECFCs from different donors, with a range of expression from 12- 78% (Fig. 2D). As for HUVECs, the expression was also variable ranging from 26 to-59% at different passages and confluencies (Fig. 2D). No significant statistical difference was observed between these cell types (One-way ANOVA, p value 0.3780).

### Kinetics of CD34 expression in the sorted cells

**Figure 3:**
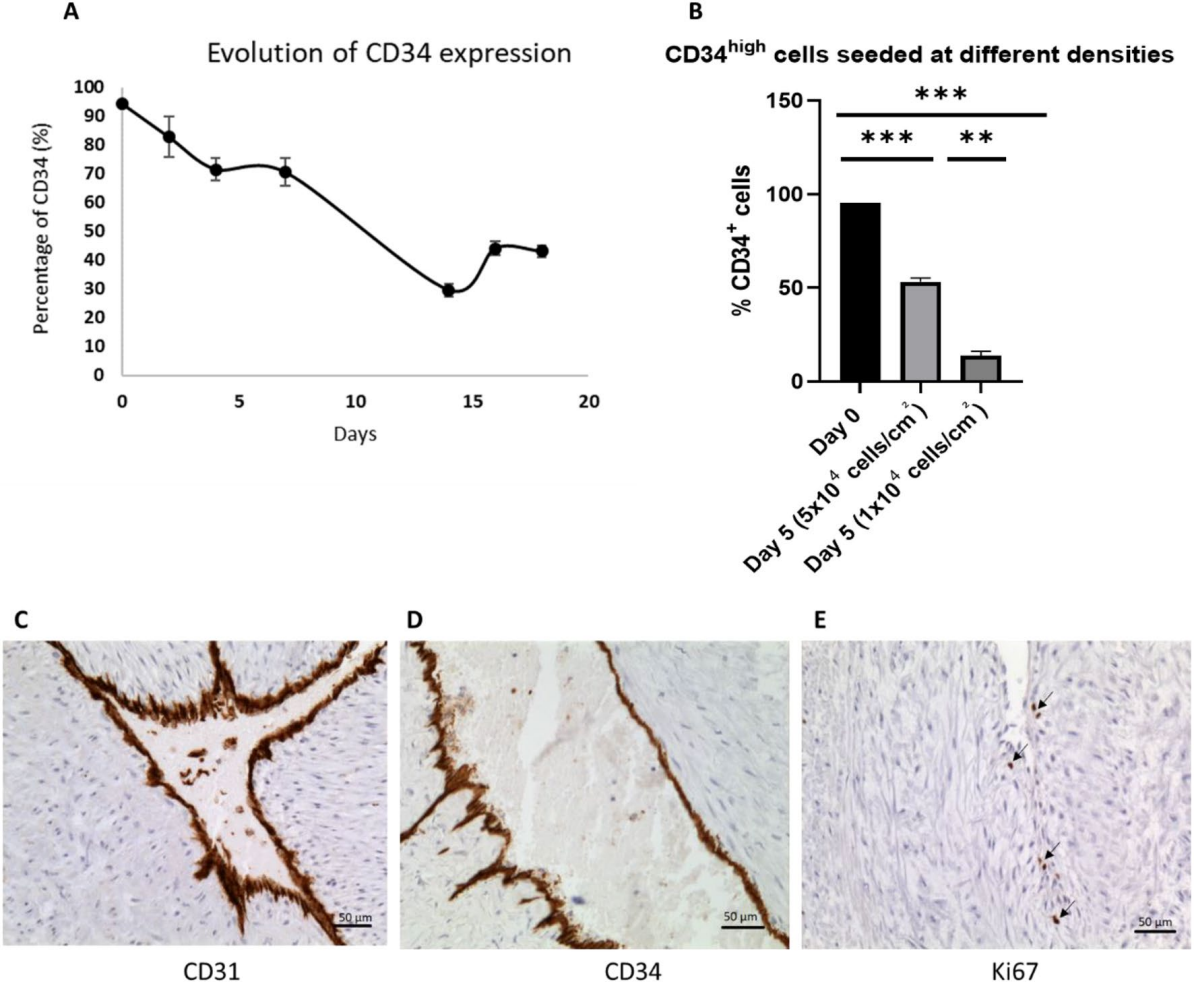
Kinetics of CD34 expression over time. CD34 sorted HUVECs were reseeded at a density of 2 × 10^4^ cells/cm^2^ and CD34 expression was monitored over time (**A**). To evaluate the effect of confluence and cell proliferation on CD34 expression, magnetically sorted CD34^high^ HUVECs (**B**) were seeded at a high density of 5 × 10^4^ cells/cm^2^ or 1 × 10^4^. CD34 expression was evaluated by flow cytometry. Endothelial markers were studied by immunostaining of the umbilical cord vein. In physiological conditions, all endothelial cells expressed CD31 (**C**) and CD34 (**D**). In the umbilical cord, very few cell were Ki67 positive and proliferating. These cells were at the periphery of the endothelium (**E**).

Sorted HUVECs were seeded at a density of 2 × 10^4^ cells/cm^2^ and CD34 expression was monitored over time. The results showed that in CD34^high^ cells, expression dropped over time and increased again after day 16 to about 40% (Fig. 3A). However, the percentage never attained the initial high level (92% immediately after sorting versus 37. 2 ± 3.38% after day 18 of reseeding), showing the unstable and partially reversible expression of this marker in-vitro.

In a second experiment, HUVECs were sorted and CD34^high^ and CD34^low^ cells were seeded at a density of 5 × 10^4^ or 1 × 10^4^ cells/cm^2^ and CD34 expression was evaluated at day 5. Results demonstrated that cells reseeded at a high density preserved a much higher expression of CD34 compared to lower densities where cells completely lose CD34 expression (Fig. 3B).

CD34^+^ expression was analyzed, ex vivo*,* by immunohistology of umbilical cord blood veins. The results showed that all HUVEC were positive for CD31 and CD34 (Fig. 3C,D). Staining with Ki67 revealed that very few cells in the umbilical cord were positive, and were outside the endothelium, suggesting little proliferation under these conditions (Fig. 3E).

### Comparative transcriptomic analysis

**Figure 4:**
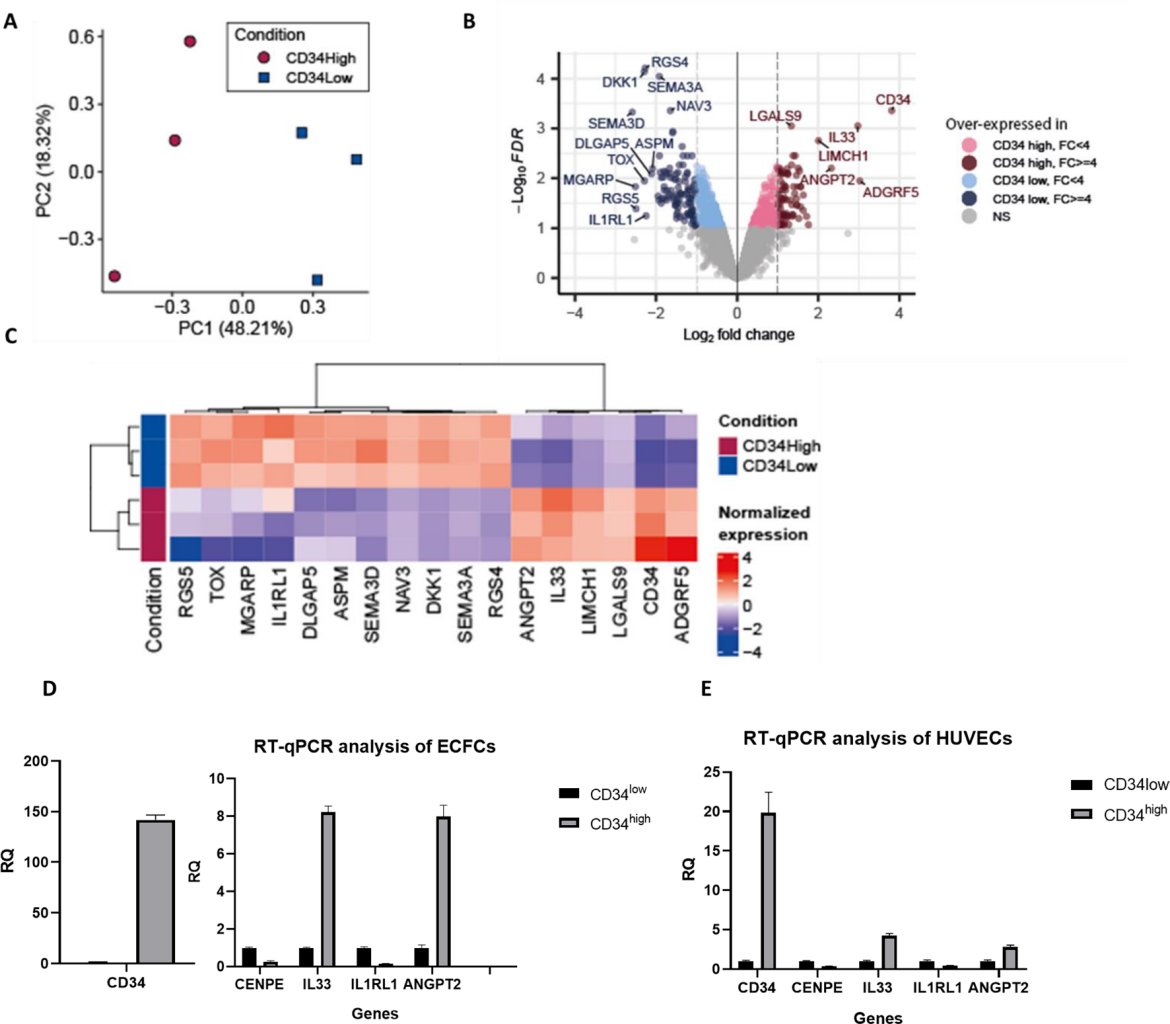
PCA mapping of the transcriptomic results, showing two distinct classes of cells based on the expression of CD34 (**A**). The results are represented in a volcano plot (adjusted *p* value vs. log2(fold change)) (**B**). Hierarchical clustering of the genes represented as heatmap (**C**). Figures B and C represent the genes which fulfill the following criteria: adjusted *p* value < 0.1 and Fold Change (FC) >  = 4 or <  = 0.25. Several genes (CD34, IL-33, IL-1RL1 and ANGPT2) identified by the transcriptomic results were validated by RT-qPCR in ECFCs (**D**) and HUVECs (**E**), after a magnetic cell sorting.

PCA mapping of the transcriptomic results identified two distinct groups of cells, based on their expression of CD34 (Fig. 4A). Transcriptomic results revealed a differential expression of 498 coding genes by at least twofold (Fig. 4B, Supplementary Table 1). Among these genes, CD34 was upregulated by a factor of 14.042 in CD34^high^ ECFCs, revealing strong correlation between gene and protein expression (Fig. 4C, Supplementary Table 1).

To understand the implication of differentially expressed genes in cell signaling pathways, they were then analyzed with Wikipathways. Many genes were involved in cell cycle progression and cell division, among which AURKA, KIF18A, KIF 20A, CENPE, MKi67. All these genes were strongly downregulated in CD34^high^ cells compared to CD34^low^ ones (Supplementary Table 1).

Other differentially expressed genes included soluble factors. As an example, IL-33 and ANGPT2 were overexpressed in CD34^high^ compared to CD34^low^ cells, by a factor of 7.866, adjusted p value 0.001; and 5.006, adjusted *p* value 0.006 respectively (Supplementary Table 1) whereas IL1RL1 (coding for ST2, the receptor of IL-33) was downregulated by a factor of 4.7, adjusted *p* value 0.056 (Supplementary Table 1).

These results were validated by RT-qPCR on sorted ECFCs and HUVECs to understand whether this profile was limited to only one endothelial type or was more general. The results showed that CD34 was strongly upregulated in CD34^high^ cells in both endothelial models, confirming a strong correlation between gene and protein expression. Other genes including proliferation marker CENPE, as well as IL-33 and Angiopoietin 2 (ANGPT2) were upregulated, while IL1RL1 was downregulated in CD34^high^ cells compared to CD34^low^ ones. The results were similar in HUVECs and ECFCs (Fig. 4D,E), thereby validating the results obtained by the transcriptomic study.

### CD34^high^ ECs secreted high levels of IL-33 and Angiopoietin 2

Because expression of ST2, IL-33 and Angiopoietin 2 was different in CD34^high^ compared with CD34^low^ cells, secretion of these soluble factors by the sorted cells, alone or in interactions with PBMCs, was evaluated.

**Figure 5:**
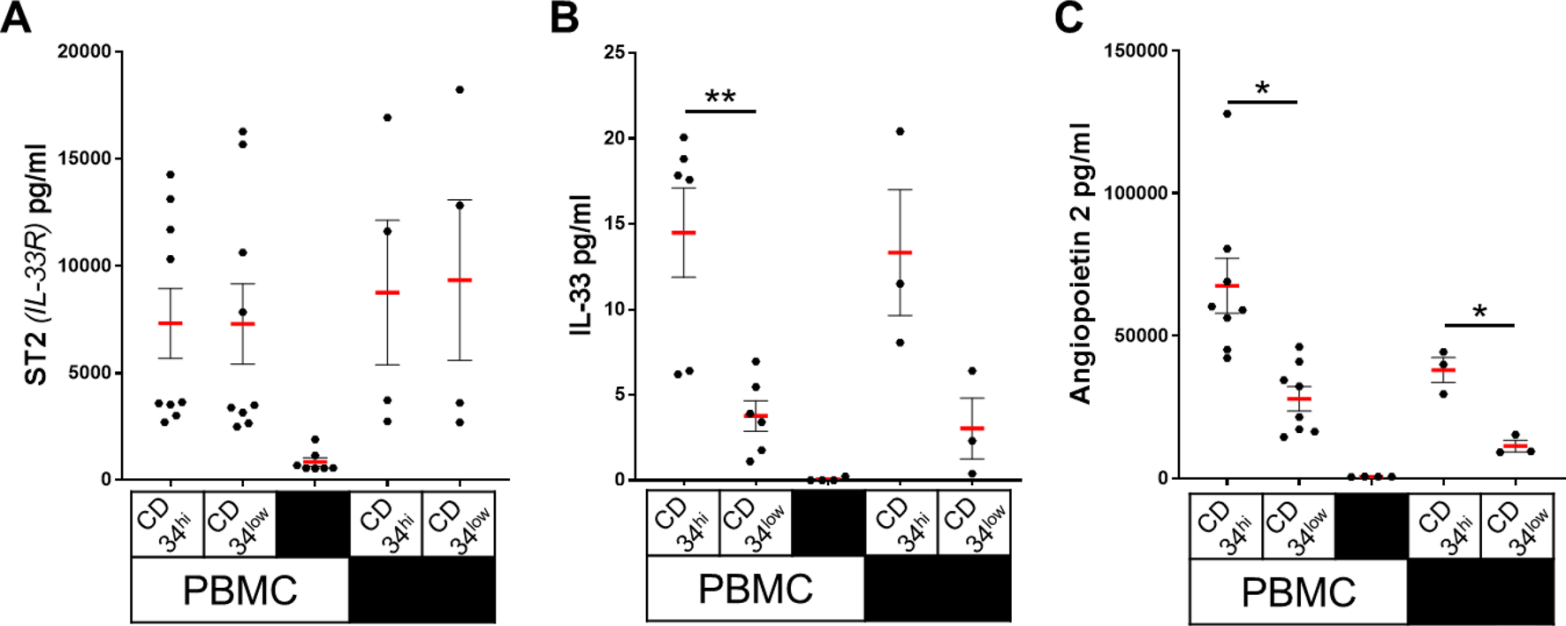
ST2 (IL-33R), IL-33 and Angiopoietin 2 secretion by CD34 sorted HUVECs. CD34^high^ and CD34l^ow^ HUVECs were seeded at a high confluence and after adhesion, were cultured alone or in co-culture with PBMCs. Secretion of ST2 (IL-33R) (**A**), of IL-33 (**B**) and of Angiopoietin 2 (**C**) was quantified at day 4. PBMC alone were used as a negative control. Each dot corresponds to an experiment and thick, horizontal lines represent median values (*p < 0.05, **p < 0.01: two-tailed paired T test).

Our results revealed ST2 concentrations of about 9000 pg/ml at day 5 (8758 ± 3374 pg/ml for CD34^high^ and 9340 ± 3745 pg/ml for CD34^low^). The concentrations were similar between both subpopulations, with or without co-culture with PBMCs (7322 ± 1632 pg/ml for CD34^high^ and 7293 ± 1877 pg/ml for CD34^low^) (Fig. 5A).

Unlike ST2, a major difference in IL-33 secretion was observed between CD34^high^ and CD34^low^ cells. IL-33 secretion was much stronger in CD34^high^ than by CD34^low^ cells (day 5 : 13.3 ± 3.7 pg/ml for CD34^high^ and 3 ± 1.8 pg/ml for CD34^low^). These results were similar between HUVECs alone or in co-culture with PBMCs (day 5: 14.5 ± 2.6 pg/ml for CD34^high^ and 3.8 ± 0.9 pg/ml for CD34^low^). Only very low levels of these factors were detected in supernatants of PBMCs alone. (Fig. 5B).

Secretion of Angiopoietin 2 was also analyzed under these conditions. Similar to data obtained from the transcriptomic analysis, a significantly higher level of Angiopoietin 2 protein was observed in cultures of CD34^high^ (37,970 ± 7580 pg/ml) compared with CD34^low^ cells (11,330 ± 3490 pg/ml). Comparable results were obtained in co-cultures with PBMCs: 67,535 ± 27,266 pg/ml in CD34^high^ cells versus 27,927 ± 12,131 pg/ml in CD34^low^ cells. It should be noted, however, that secretion levels were considerably higher in co-cultures with PBMCs compared to HUVECs alone (Fig. 5C).

### CD34 expression and EC allogenicity:

Under inflammatory conditions, ECs selectively amplify allogeneic CD4^+^-T responses leading to differentiation of Th1, Th17 and Treg sub-populations. We examined whether CD4^+^-T differentiation was modified by the level of CD34 expression on ECs. HUVECs were pretreated with IFNγ prior to cell sorting. This treatment did not alter CD34 expression (data not shown) but increased HLA-DR and CD54 as previously reported^20^.

**Figure 6:**
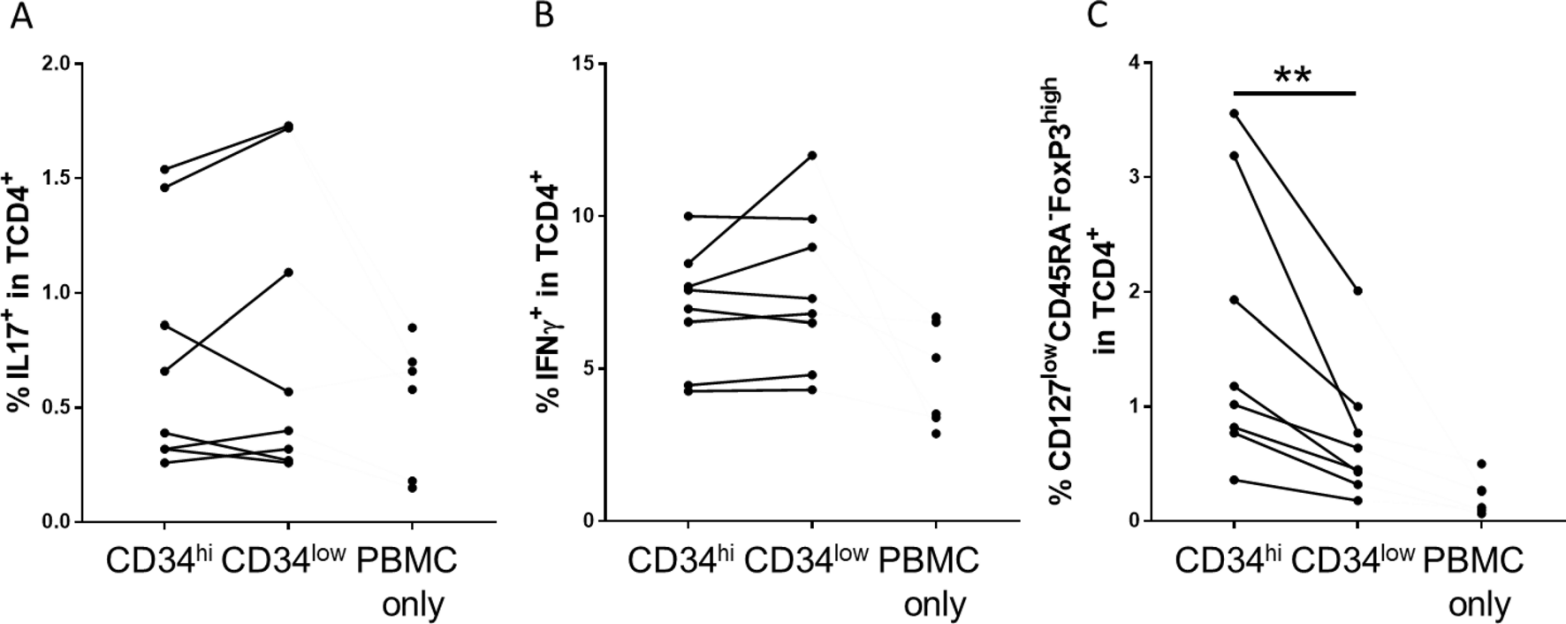
CD4^+^ lymphocyte polarization, induced by CD34 sorted HUVECs. CD34^high^ and CD34^low^ HUVECs were seeded at a high confluence and after adhesion, they were co-cultured with PBMCs. Percentage of different CD4^+^ T lymphocytes, including Th17 (IL-17^+^ cells in CD4^+^T cells) (**A**), Th1 (IFNγ^+^ cells in CD4^+^T cells) (**B**), and Tregs (% CD127^low^CD45RA^−^FoxP3^high^ in CD4^+^T cells) (**C**) was evaluated at day 4 (**C**) and day 5 (**A**,**B**). PBMC alone (PBMC only) were used as a control. The results are shown as before and after representation and each dot correspond to an experiment (*p < 0.05, **p < 0.01: two-tailed Wilcoxon test).

PBMCs co-cultured with sorted HUVECS were evaluated by flow cytometry at day 4 for Tregs and on day 5 for Th1/Th17 sub-populations. The proportions of either Th17 or Th1 (Fig. 6A,B) cells were similar in the presence of CD34^high^ or CD34^low^cells. However, the proportion of Treg, identified as CD127^low^ CD45RA^−^FoxP3^high^ cells in the T-CD4^+^ population was two-fold higher in co-cultures with CD34^high^ (1.6 ± 0.4%) compared with CD34^low^ (0.73 ± 0.2%) HUVECs (Fig. 6C).

These results indicate that CD34 expression by ECs is associated with a higher capacity to differentiate Tregs in EC-dependent allogeneic responses.

### Mechanisms of Treg differentiation by CD34^high^ ECs

**Figure 7:**
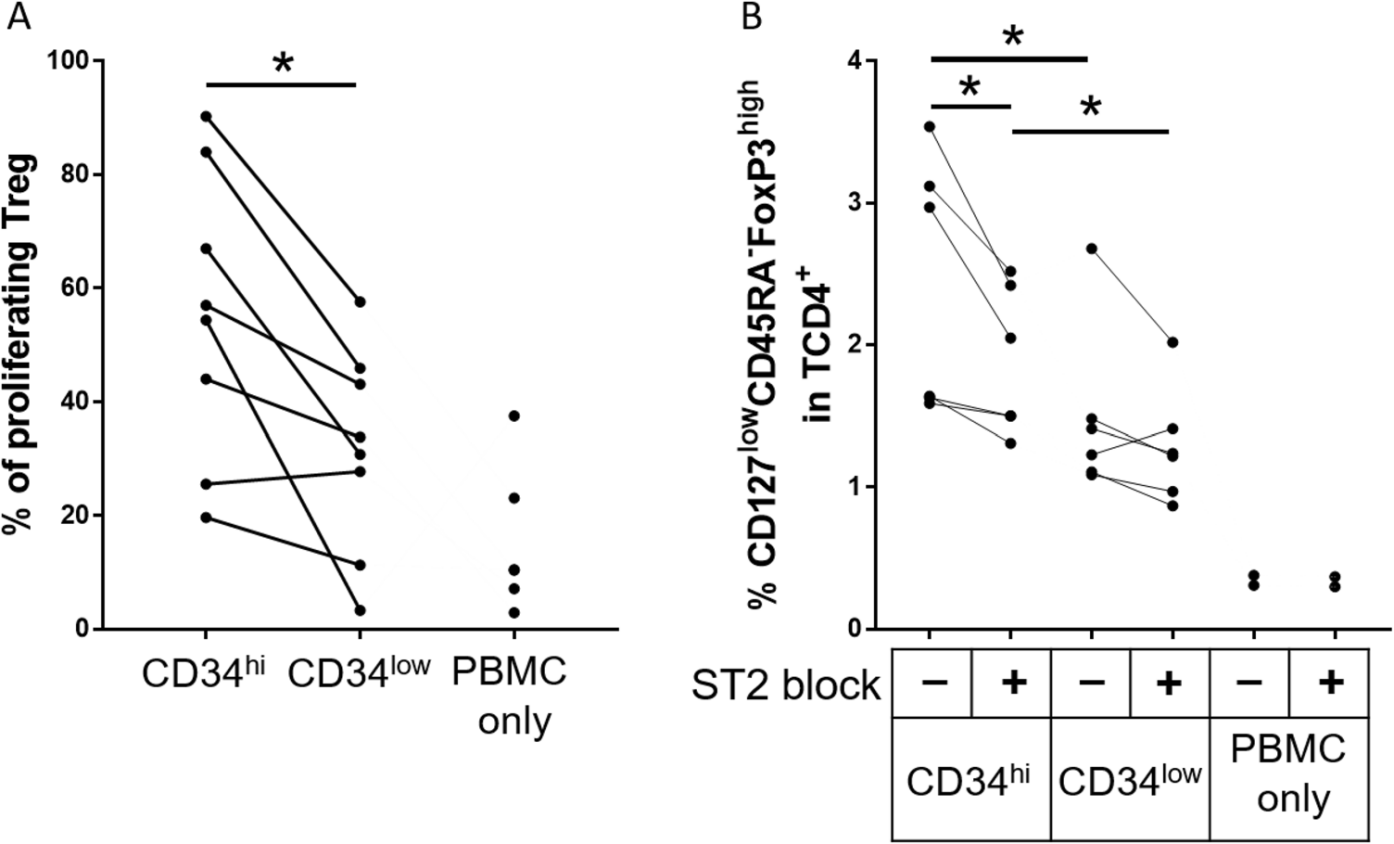
Mechanisms of Treg induction by CD34 sorted HUVECs. CD34^high^ and CD34^low^ HUVECs were seeded at high confluence and after adhesion, were co-cultured with PBMCs for 4 days (**A**) or before co-culture, HUVECs and PBMC were pre-treated with an antibody directed against the IL-33R (ST2) to block IL-33 interaction with its receptor (**B**). Proliferation of Treg (% of CFSE- cells in Treg population) is also shown (**A**). The % Treg (% CD127^low^CD45RA^−^FoxP3^high^ in CD4^+^T cells) (**B**) was evaluated at day 4. PBMC alone (PBMC only) were used as a control. The results are shown as before and after representation and each dot correspond to a single experiment (*p < 0.05, **p < 0.01: two-tailed Wilcoxon test).

In a model of allogeneic PBMCs interaction with microvascular ECs, we previously reported that amplification of the Treg population is partially dependent upon Treg proliferation^21^. We therefore determined whether Tregs were proliferating in co-cultures of CD34^high^ ECs with allogeneic PBMCs. As shown in Fig. 7A and Supplementary Figure 1, the proportion of proliferating Treg was significantly higher in co-cultures with CD34^high^ compared to CD34^low^ EC (55 ± 9% of proliferating Treg with CD34^high^ and 32 ± 6% with CD34^low^, *p* = 0.0156).

It has been reported in the mouse that the interaction between IL-33 and ST2 is involved in colonic Treg amplification^22^.We therefore examined whether inhibition of the IL-33 and ST2 interaction altered the ability of CD34^high^ cells to differentiate allogeneic Tregs. The interaction was inhibited by addition of a ST2 blocking antibody. Under these conditions, the proportion of Tregs amplified by both CD34^high^ and CD34^low^ ECs was decreased (Fig. 7B), thus suggesting that ST2 is implicated in Treg differentiation by EC and that inhibition of the ST2 and IL-33 interaction reduces the Treg population activated by CD34^high^EC (1.9 ± 0.2% and 2.4 ± 0.4% of CD127^low^CD45RA-FoxP3^high^ in CD4^+^T cells for CD34^high^ cells respectively with or without ST2 blocking compared to 1.3 ± 0.2% and 1.5 ± 0.3% for CD34^low^ cells respectively with or without ST2 blocking). It should be noted that we observed an accumulation of IL-33 in the co-culture supernatant in the presence of a ST2 blocking antibody (Supplementary Figure 2) suggesting an efficient blocking, resulting in the absence of binding and consumption of IL-33, the ligand for ST2.

## Discussion

CD34 is one of the endothelial markers that is highly regulated in cell culture models in-vitro and in pathological conditions in-vivo. Our transcriptomic study on ECFCs, as well as our RT-qPCR results both on ECFCs and HUVECs confirmed that CD34^high^ cells had increased expression of CD34 and Angiopoietin 2 genes. Our results were similar to those described previously on HUVECs by Siemerink et al*.*^16^. However, the current study also revealed an overexpression of IL-33 in CD34^high^ compared to CD34^low^ cells. The qPCR results were validated both in ECFCs and HUVECs, suggesting a similar and general in-vitro behavior of different endothelial models, affected by culture conditions rather than by their source of origin.

To confirm the effect of confluence on EC proliferation and CD34 expression, sorted HUVECs were seeded at higher (5 × 10^4^ cells/cm^2^) or lower densities (1 × 10^4^ cells/cm^2^). At day 5, highly confluent CD34^high^ cells maintained 50% of CD34 expression whereas at lower confluence, only 16% of cells maintained this expression. When allowed to achieve very high confluence, CD34 expression was partially restored in both CD34^high^ cells that then lost expression after re-seeding. These results suggest that the in-vitro CD34 expression on ECs is partially regulated by their confluence and by low levels of proliferation, and that expression is unstable and reversible. It would be interesting to understand the mechanisms of regulation of CD34 expression by cell proliferation and confluence. It has been previously reported that up-regulation of surface CD34 is associated with protein kinase C (PKC) induced hyperphosphorylation of this molecule in hematopoietic cells^23^. PKC is also an important element regulating cell cycle progression^24^. Therefore, CD34 expression could be partly influenced by post-transcriptional modifications induced by PKC. Our transcriptomic study showed that many kinases are downregulated in CD34^high^ cells, including protein kinase C alpha (PRKCA), NIMA-related kinase 7 (NEK7) and polo-like kinase 1 (PLK1) which are all involved in cell cycle progression (Supplementary Table 1). Our results also indicate a direct correlation between CD34 gene and protein expression. Therefore, it will be important to understand the transcriptional regulation of CD34 in ECs and to compare in-vitro with in-vivo mechanisms involved in vascular development.

Another important difference between CD34^high/low^ cells concerned their ability to secrete soluble factors, notably IL-33. This cytokine belongs to the IL-1 family and is constitutively expressed in the nuclei of endothelial and epithelial cells^25^. It acts through binding to its receptor ST2, coded by IL1RL1 gene that may be expressed in a soluble or a membrane-bound forms^26^.

In our study, the overexpression of IL-33 in CD34^high^ cells was confirmed both at the transcriptional and protein levels. However, both CD34^high^ and CD34^low^ secreted ST2 at similar concentrations, despite differences observed at transcriptional level. Only soluble ST2 secretion was quantified. It is possible that ST2 membrane expression is different between CD34^high^ and CD34^low^ cells. None of these factors were significantly detected in cultures of PBMCs alone, suggesting that they are mainly secreted by ECs. Co-culture of PBMCs and sorted HUVECs showed that Th1 and Th17 cells expanded equally in the presence of CD34^high/low^ HUVECs whereas both Treg polarization and proliferation were significantly higher in the presence of CD34^high^ ECs.

A possible mechanism for the regulation of Treg proliferation by CD34^high^ cells may involve the higher secretion of IL-33 by these cells. Our study reveals that blocking the interaction between IL-33 and its receptor reduced the expansion of Treg, thus demonstrating the importance of IL-33 in the regulation of the Treg population in this context. The role of IL-33 in immune cell activation and differentiation is currently controversial. It has been reported that IL-33 acts as an alarmin, released by damaged ECs and associated with pro-inflammatory and allergic reactions^27^. In contrast, other studies suggest that under inflammatory conditions, IL-33 is implicated in the regulation of immune responses. As an example, after mice exposure to lung allergens, IL-33 released activated Treg to suppress γδ T cell responses^28^.

Another cytokine which was overexpressed in CD34^high^ ECs was Angiopoietin 2. This has been previously shown by transcriptomic analysis^16^. In the current study, we have confirmed previous results by transcriptomic assay as well as by protein secretion. The previous study concluded that Angiopoietin 2 was related to endothelial “tip cells”^16^. However, the immunological role of this cytokine was not discussed. Regarding Treg differentiation, it has been reported that Angiopoietin 2 stimulates the release of IL-10 by TIE2-expressing monocytes/macrophages and thus indirectly promotes Tregexpansion^29^. Therefore, understanding mechanisms implicating CD34 and Angiopoietin 2 in Treg expansion is another axis that requires exploration.

In conclusion, we have demonstrated an association between CD34 expression, quiescence, IL-33 secretion, Treg expansion and regulation of Treg proliferation by ECs in-vitro. However, the molecular mechanisms relating these observations remain to be fully elucidated.

Unlike previous studies that linked CD34 expression to endothelial “stem cells” or “tip cells”, we propose that it may be a marker of less activated, more mature and an anti-inflammatory state of ECs in-vitro. In this study, we also considered the regulation of T-CD4^+^ lymphocytes by ECs. In future studies, it will be important to take into consideration other populations such as innate immune cells, these should also be evaluated in co-culture models with ECs.

Finally, it would be interesting to examine endothelial CD34 expression in *ex-vivo* samples from different pathologies, such as in liver fibrosis^30^. Understanding the mechanisms of CD34 expression could pave the way to target the endothelium for therapeutic purposes.

### Supplementary Information

Supplementary Table 1.

Supplementary Figures.

## Data Availability

Data and analyzed results related to the transcriptomic study can be found in the Supplementary Table 1 (CD34High_VS_CD34Low_coding_gene) Raw transcriptomic data can be found in a GEO format, with the accession number GSE236137. https://www.ncbi.nlm.nih.gov/geo/query/acc.cgi?acc=GSE236137. Other datasets used and/or analyzed during the current study are available from the corresponding author on reasonable request.
