# Supplementary material for: Endothelial CD34 expression and regulation of immune cell response in-vitro
Source: Sci Rep. 2023 Aug 19;13:13512. doi: 10.1038/s41598-023-40622-7 (PMC10439936; doi:10.1038/s41598-023-40622-7)
Supplement: Supplementary file 2 — Supplementary Figures. [file 41598_2023_40622_MOESM2_ESM.pdf]

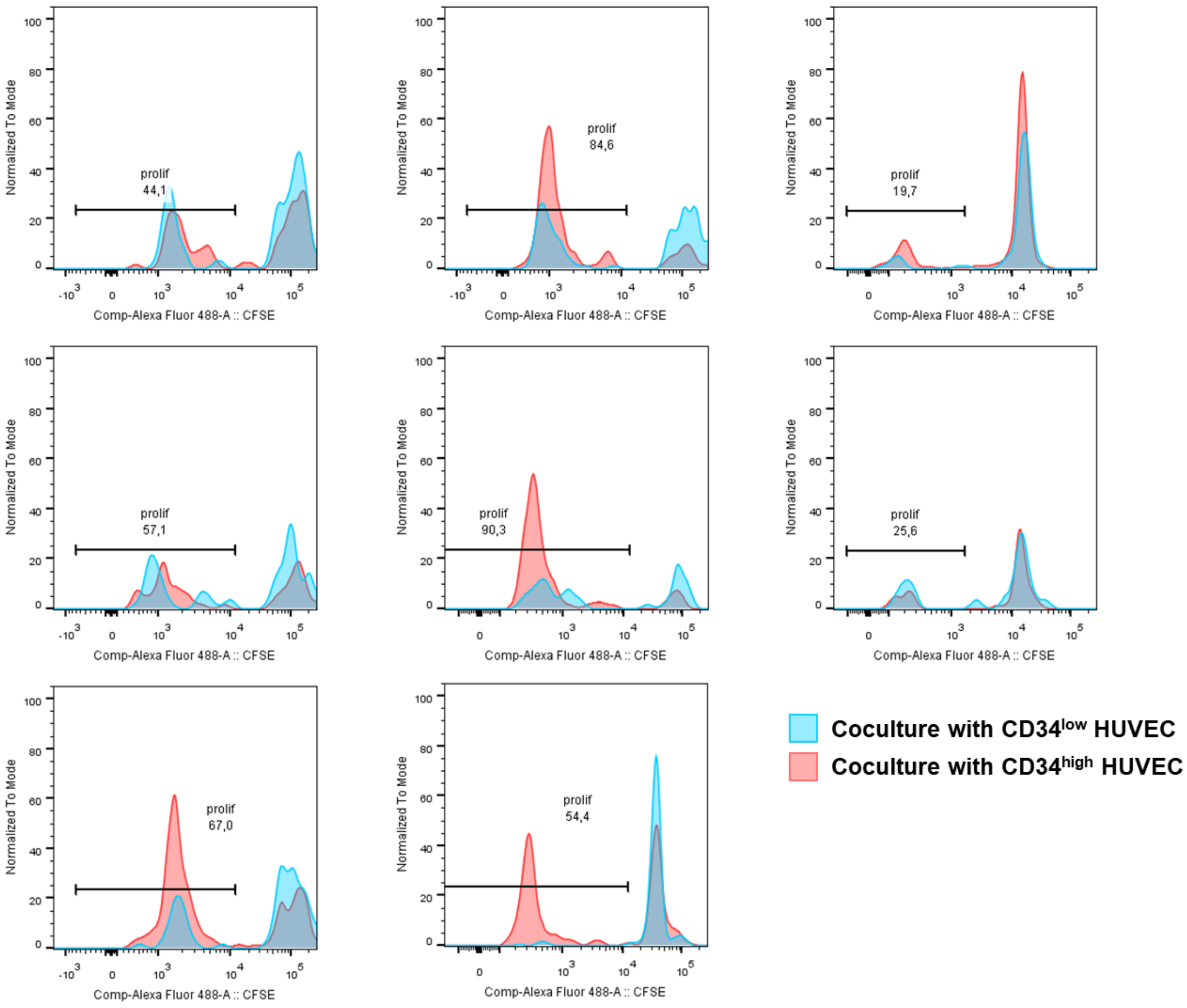

Supplementary Figure 1: Histogram representation of T-reg proliferation percentage, after 4 days of PBMC co-culture with $CD34^{low}$ or $CD34^{high}$ HUVEC. The percentage was defined by CFSE dilution.

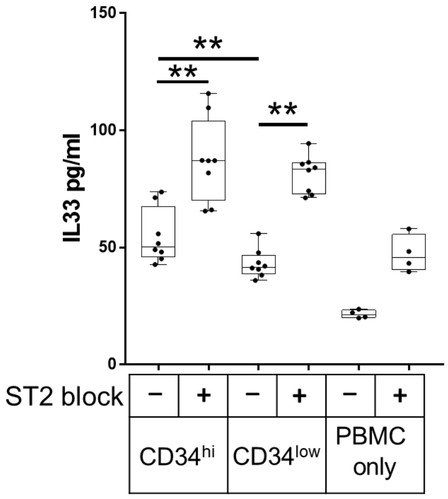

Supplementary Figure 2: ST2 blocking leads to IL-33 accumulation. CD34high and CD34low HUVECs were seeded at a high confluence and after adhesion, HUVECs were pre-treated as PBMCs with an antibody directed against the IL-33R (ST2) to block IL33 interaction with its receptor before culture. After 4 days, secretion of IL-33 was quantified in the supernatant of co-culture. PBMC alone (PBMC only) were used as a control. Data were represented by box and whiskers (Min to Max) (**p < 0.01, Wilcoxon test). Each dot corresponds to an experiment.
